# The impact of temporal trends in stroke event rates and 30-day case fatality on trends in stroke mortality: an international comparative study

**DOI:** 10.1093/ije/dyag157

**Published:** 2026-09-18

**Authors:** Lee Nedkoff, F Lucy Wright, Nhung Nghiem, Solmaz Setayeshgar, Emmalin Buajitti, Aden C H Kwok, Dawit Zemedikun, Ximena Camacho, Samantha Stiles, Raphael Goldacre, Kate Smolina, Laura C Rosella, Tony Blakely, Philip Clarke

**Affiliations:** Cardiovascular Epidemiology Research Centre, School of Population and Global Health, The University of Western Australia, Crawley, Western Australia, Australia; Cardiology Population Health Lab, Victor Chang Cardiac Research Institute, Sydney, NSW, Australia; Applied Health Research Unit, Nuffield Department of Population Health, and the NIHR Oxford Biomedical Research Centre, University of Oxford, Oxford, United Kingdom; John Curtin School of Medical Research, Australian National University, Canberra, Australia; Department of Public Health, University of Otago, Wellington, New Zealand; Epidemiology and Intelligence Services, BC Centre for Disease Control, Vancouver, BC, Canada; Dalla Lana School of Public Health, University of Toronto, Toronto, ON, Canada; Applied Health Research Unit, Nuffield Department of Population Health, and the NIHR Oxford Biomedical Research Centre, University of Oxford, Oxford, United Kingdom; Cardiovascular Epidemiology Research Centre, School of Population and Global Health, The University of Western Australia, Crawley, Western Australia, Australia; Cardiology Population Health Lab, Victor Chang Cardiac Research Institute, Sydney, NSW, Australia; School of Population Health, Faculty of Medicine and Health, University of New South Wales, Sydney, New South Wales, Australia; Cardiovascular Epidemiology Research Centre, School of Population and Global Health, The University of Western Australia, Crawley, Western Australia, Australia; Applied Health Research Unit, Nuffield Department of Population Health, and the NIHR Oxford Biomedical Research Centre, University of Oxford, Oxford, United Kingdom; Epidemiology and Intelligence Services, BC Centre for Disease Control, Vancouver, BC, Canada; Dalla Lana School of Public Health, University of Toronto, Toronto, ON, Canada; Population Interventions Unit, Centre for Epidemiology and Biostatistics, Melbourne School of Population and Global Health, University of Melbourne, Parkville, Australia; Health Economics Research Centre, Nuffield Department of Population Health, University of Oxford, Headington, United Kingdom

**Keywords:** stroke, mortality, case fatality, event rates, trends, relative contributions

## Abstract

**Background:**

Stroke mortality rates have declined in recent decades; however, data on contemporary drivers of this trend are limited. This study compared the contribution of trends in stroke event rates and 30-day case fatality to declines in stroke mortality from 2002 to 2018.

**Methods:**

Linked hospitalization and mortality data were obtained from Western Australia (WA), England, New Zealand, British Columbia (BC), and Ontario. Age-standardized and age-specific stroke mortality and event rates and 30-day case fatality were calculated and age-adjusted trends estimated from Poisson and binomial regression models. The relative contribution of trends in stroke events and 30-day case fatality to trends in stroke mortality were calculated.

**Results:**

There were 2 248 242 stroke events identified. Age-adjusted reductions in event rates ranged from −0.96%/year [95% confidence interval (CI) − 1.22, 0.70%] in men in WA to −2.51%/year (95% CI −2.61, −2.40%) in women in Ontario. Age-standardized 30-day case fatality was highest in England in 2002 (men 43.7%, women 45.5%), but declined at a greater rate than in other jurisdictions. Declines in stroke mortality ranged from −3.3%/year to −5.5%/year. Reductions in 30-day case fatality were the major contributor to declines in stroke mortality [range: men, 63.5% (Ontario)–80.9% (England); women, 55.3% (BC)–73.1% (England)]. In people aged <65 years, stroke mortality declines were almost completely driven by reductions in 30-day case fatality with increasing or attenuated event rates among those aged 35–64 years.

**Conclusion:**

Improvements in 30-day case fatality had a greater impact on stroke mortality than trends in event rates, suggesting that further gains could be achieved with targeted prevention. Adverse trends in event rates in people aged <65 years could signal future increases in stroke mortality.

Key Messages

- This study aimed to measure the epidemiologic drivers of trends in stroke mortality by measuring the relative contribution of trends in stroke event rates and 30-day case fatality to declines in stroke mortality by jurisdiction, age, and sex.
- Stroke mortality has substantially reduced in these five jurisdictions, underpinned by improvements in 30-day case fatality, with age-specific analyses showing that, in those aged <65 years, reductions in 30-day case fatality are the key drivers of improvements in mortality due to limited or no improvement in event rates.
- There are very limited data measuring contributions of event rates and case fatality to trends in stroke mortality, and understanding these patterns will inform targets for further reducing stroke mortality and averting adverse trends in mortality in younger age groups.

## Introduction

Stroke remains a leading cause of death and disability worldwide. In 2021, stroke was responsible for 160 million disability-adjusted life years (DALYs), making it the fourth-most common cause of DALYs and indicating its high population burden [1]. There have been consistent declines in stroke mortality in high- and middle-income countries over the past three decades, concurrently with advances in stroke treatment and reductions in the prevalence of key risk factors [1]. Understanding the factors contributing to reductions in stroke mortality is essential to inform targeted prevention and treatment.

Reductions in stroke event rates and case fatality are suggested as drivers of declining stroke mortality [2, 3], although there are limited data on the specific contribution of each. The multinational WHO MONICA study showed that reductions in stroke event rates and case fatality were concurrent with declines in stroke mortality between 1985 and 1995 [4], while a more recent English study demonstrated that declining 30-day case fatality was a bigger contributor to declining stroke mortality than reductions in event rates [5]. While early case fatality following stroke hospitalization has improved substantially [6, 7], around one-third of stroke deaths occur in the prehospital setting, indicating the importance of also understanding this burden [3].

Many studies from high-income countries have reported reductions in stroke event rates [8–10], despite changing stroke diagnostic criteria [11]. There is also evidence of differing trends in stroke event rates by age [8, 12], with a meta-analysis of stroke incidence studies demonstrating suboptimal temporal trends in younger compared with older adults [13]. Reducing the burden of stroke therefore relies on understanding the drivers of stroke trends to identify where interventions can be most effective.

Comparative studies between countries can provide effective benchmarking of trends and increased understanding of stroke burden. We have previously undertaken this approach for myocardial infarction (MI) in jurisdictions with high-quality linked health data [14]. Using linked health data from England, New Zealand, Western Australia (WA), and British Columbia (BC) and Ontario (Canada), we compared temporal trends in stroke event and mortality rates and 30-day case fatality between jurisdictions from 2002 to 2018; estimated the relative contribution of trends in stroke event rates and 30-day case fatality to trends in stroke mortality; and determined whether burden, trends, and contributions differed by age and sex.

## Methods

### Data sources and study population

We used person-linked hospitalization and mortality records, with data linkage and extraction undertaken by the relevant health departments, a federated model and common analysis plan, and the pooling of aggregated data by the coordinating centre (University of Western Australia). Hospitalizations and deaths in England were recorded by using the WHO International Classification of Diseases tenth revision (ICD-10), with country-specific modifications (BC, Ontario: ICD-10-Canada; WA, New Zealand: ICD-10-Australian Modification for hospitalizations), hereafter termed ICD-10. Records with missing or invalid values for sex, age, or admission or death dates were excluded (≤0.2% in WA, England, BC, New Zealand; Ontario, no missing data).

The available dataset in each jurisdiction included all hospitalizations and deaths for cerebrovascular disease (ICD-10 I60-I69) from 1 January 2002 to 31 December 2018, representing the maximum data available across jurisdictions and more homogeneous healthcare prior to the COVID-19 pandemic. We investigated patterns in the coding of stroke in hospital and mortality data to inform study parameters (Supplementary File S1 and Supplementary Figures 1–3).

### Event definitions

We included all people aged ≥35years with a stroke hospitalization or death coded as intracerebral haemorrhage (ICD-10 I61), other non-traumatic intracranial haemorrhage (I62), cerebral infarction (I63), or unspecified stroke (I64). Subarachnoid haemorrhage (I60) was excluded due to differing aetiology and management, aligning with previous studies [5]. We did not stratify by type of stroke, as >50% of stroke deaths were classified as unspecified (Supplementary Figure 3).

We defined a ‘stroke death’ as being where stroke was recorded as the underlying cause of death. ‘Sudden stroke deaths’ were stroke deaths with no stroke hospital admission in the prior 30 days. ‘Stroke hospital admissions’ were those in which stroke was the principal discharge diagnosis. To reduce the double-counting of inter/intra-hospital transfers and early readmission for the same event, stroke admissions occurring within 30 days of each other were counted as one admission. ‘Stroke events’ comprised stroke hospital admissions and sudden stroke deaths. An individual could have multiple stroke admissions and/or a sudden stroke death included if each event met the specified criteria. ‘Fatal strokes’ comprised fatal stroke admissions (all-cause death ≤30 days following stroke admission) and sudden stroke deaths (Supplementary Figure 4).

### Statistical analyses

All analyses were stratified by jurisdiction, sex, and broad age group (35–54, 55–64, 65–74, 75–84, ≥85 years). Annual event and mortality rates per 100 000 person-years were calculated by using stroke events and stroke deaths, respectively, as the numerator, and annual population estimates from each jurisdiction as the denominator. Event and mortality rates were age-standardized by 5-year age groups via the direct method, with the 2013 European Standard Population as the standard. Thirty-day case fatality used fatal strokes as the numerator and stroke events as the denominator, and was age-standardized by using the age distribution of stroke events in each jurisdiction as its standard population (Supplementary Figure 5).

Temporal trends in stroke event and mortality rates were estimated by using Poisson regression models. The dependent variable was the number of events or deaths and the independent variables were 5-year age group and calendar year. The average annual changes with 95% confidence intervals (CIs) were calculated from the exponential of the β-coefficient for calendar year. Poisson models were checked for overdispersion and a dispersion parameter was included where present. Trends in case fatality were estimated by using a generalized linear model with binomial distribution and logit-link function, with fatal stroke as a binary dependent variable.

Estimation of relative contributions of trends in event rates and case fatality to trends in stroke mortality was based on published methods [5, 14, 15]. Briefly, an estimated annual trend in mortality was calculated by adding the average annual change in event rates and case fatality, and the relative contribution of each was calculated by dividing the change in event rates or case fatality by the average annual change in mortality rates. Where opposing trends resulted in summed contributions exceeding 100%, values were constrained to 0% and 100% for interpretability. We calculated 95% CIs around the relative contributions by using bootstrapping (10 000 iterations), drawing simulated values from a log-normal distribution by using the regression coefficients and standard errors. The actual relative contributions remained consistent with the bootstrapped estimates. We modelled the relative contributions on a logit scale by using ordinary least squares regression including two-way interaction terms for age group, sex, and jurisdiction, restricted to those aged ≥65 years due to opposing trends in event rates and 30-day case fatality in younger age groups.

Analyses were undertaken by using SAS (v9.4 WA, BC; Enterprise Guide 7.1 New Zealand, Ontario) and R4.4.2 and Stata v14.1 (England).

## Results

### Cohort characteristics

The total population across all jurisdictions was 84.2 million. WA had the highest proportion of people aged <65 years (85.6%), with the lowest in BC and England (81.8%). There were 2 248 242 stroke events in people aged ≥35 years, with 1 072 361 in men (47.7%) and 1 175 881 in women (52.3%) (Table 1). There was limited variation between jurisdictions in the mean age at time of stroke, with women on average 6 years older than men. In men, 84.5% of stroke events were stroke admissions versus 78.4% in women. The majority of stroke events occurred in men aged ≥75 years and in women aged ≥85 years.

**Table 1:** Descriptive characteristics of stroke events by jurisdiction and sex in people aged ≥35 years, 2002–18.

		Western Australia	England	New Zealand	British Columbia	Ontario
**MEN**						
Population size^a^ (*n*)		1 310 728	27 667 942	2 397 160	2 479 444	7 881 770
Population size^a^ aged <65 years (*n*)		1 132 725 (86.4)	23 019 247 (83.2)	2 041 250 (85.2)	2 052 563 (82.8)	6 703 573 (85.1)
Stroke deaths (*n*)		4289	207 824	11 304	12 845	26 409
Stroke events		23 025	807 663	60 231	48 427	133 015
Mean age [years (± SD)]		72.83 (13.01)	73.84 (12.50)	73.51 (12.34)	74.67 (12.67)	72.76 (12.57)
Median age (years) (Q1, Q3)		75 (64, 83)	76 (66, 83)	75 (66, 83)	77 (67, 84)	74 (63, 82)
Stroke admissions [*n* (%)]		20 325 (88.3)	675 200 (83.6)	53 634 (89.0)	39 803 (82.2)	117 207 (88.1)
Fatal strokes		5609	239 297	14 268	15 455	34 694
Mean age [years (± SD)]		79.21 (11.63)	79.00 (10.86)	78.90 (11.01)	80.02 (11.12)	78.31 (11.31)
Median age, years (Q1, Q3)		81 (74, 87)	81 (74, 87)	81 (73, 87)	82 (74, 88)	80 (72, 86)
Sudden stroke deaths [*n* (%)]		2700 (48.1)	132 463 (55.4)	6597 (46.2)	8624 (55.8)	15 808 (45.6)
Fatal stroke admissions [*n* (%)]		2909 (51.9)	106 834 (44.6)	7671 (53.8)	6831 (44.2)	18 886 (54.4)
Average time to death (days)^b^ [mean (± SD)]		8.35 (7.50)	8.11 (7.92)	8.08 (7.37)	8.35 (7.87)	7.58 (7.05)
Age groups (years) [*n* (%)]						
Stroke events						
35–54		2475 (10.7)	70 383 (8.7)	5184 (8.6)	3836 (7.9)	12 657 (9.5)
55–64		3579 (15.5)	107 611 (13.3)	8529 (14.2)	6488 (13.4)	21 370 (16.1)
65–74		5503 (23.9)	190 125 (23.5)	14 832 (24.6)	10 905 (22.5)	32 276 (24.3)
75–84		7175 (31.2)	273 473 (33.9)	19 935 (33.1)	15 498 (32.0)	42 441 (31.9)
≥85		4293 (18.6)	166 071 (20.6)	11 751 (19.5)	11 700 (24.2)	24 271 (18.2)
Fatal strokes						
35–54		269 (4.8)	8690 (3.6)	525 (3.7)	502 (3.2)	1469 (4.2)
55–64		395 (7.0)	15 803 (6.6)	1035 (7.3)	1029 (6.7)	2891 (8.3)
65–74		859 (15.3)	40 990 (17.1)	2433 (17.1)	2449 (15.8)	6164 (17.8)
75–84		2062 (36.8)	92 635 (38.7)	5346 (37.5)	5331 (34.5)	12 858 (37.1)
≥85		2024 (36.1)	81 179 (33.9)	4929 (34.5)	6144 (39.8)	11 312 (32.6)
**WOMEN**						
Population size^a^ (*n*)		1 307 064	28 309 236	2 418 320	2 531 032	7 911 935
Population size^a^ aged <65 years (*n*)		1 108 435 (84.8)	22 778 678 (80.5)	2 009 810 (83.1)	2 045 165 (80.8)	6 526 428 (82.5)
Stroke deaths (*n*)		6165	313 939	18 732	17 810	36 882
Stroke events (*n*)		21 579	905 382	64 587	48 720	135 613
Mean age [years (± SD)]		78.81 (13.04)	79.8 (11.78)	78.37 (12.56)	80.5 (12.06)	78.30 (12.42)
Median age (years) (Q1, Q3)		82 (72, 88)	82 (74, 88)	81 (72, 87)	83 (74, 89)	81 (71, 87)
Stroke admissions [*n* (%)]		17 514 (81.2)	704 323 (77.8)	52 201 (80.8)	35 806 (73.5)	111 677 (82.3)
Fatal strokes (*n*)		7653	355 314	22 317	20 521	46 403
Mean age [years (± SD)]		84.71 (10.04)	83.95 (9.56)	83.89 (9.95)	85.05 (9.88)	83.34 (10.36)
Median age (years) (Q1, Q3)		87 (80, 91)	85 (80, 90)	86 (80, 90)	87 (81, 92)	85 (79, 90)
Sudden stroke death [*n* (%)]		4065 (53.1)	201 059 (56.6)	12 386 (55.5)	12 914 (62.9)	23 936 (51.6)
Fatal stroke admission [*n* (%)]		3588 (46.9)	154 255 (43.4)	9931 (44.5)	7607 (37.1)	22 467 (48.4)
Average time to death^b^ (days) [mean (SD)]		8.34 (7.15)	8.7 (8.13)	8.36 (7.41)	8.43 (7.85)	7.46 (6.91)
Age groups (years) [*n* (%)]						
Stroke events						
35–54		1512 (7.0)	41 612 (4.6)	3987 (6.2)	2033 (4.2)	7778 (5.7)
55–64		1661 (7.7)	56 716 (6.3)	5370 (8.3)	3326 (6.8)	11 494 (8.5)
65–74		3303 (15.3)	133 933 (14.8)	10 212 (15.8)	6944 (14.3)	22 772 (16.8)
75–84		6864 (31.8)	310 622 (34.3)	21 087 (32.6)	14 961 (30.7)	45 237 (33.4)
≥85		8239 (38.2)	362 499 (40)	23 931 (37.1)	21 456 (44.0)	48 332 (35.6)
Fatal strokes						
35–54		160 (2.1)	5496 (1.5)	459 (2.1)	311 (1.5)	990 (2.1)
55–64		210 (2.7)	9787 (2.8)	696 (3.1)	588 (2.9)	1756 (3.8)
65–74		589 (7.7)	31 949 (9)	1974 (8.8)	1631 (7.9)	4663 (10)
75–84		2175 (28.4)	113 573 (32)	6579 (29.5)	5565 (27.1)	14 328 (30.9)
≥85		4519 (59)	194 509 (54.7)	12 609 (56.5)	12 426 (60.6)	24 666 (53.2)

Stroke deaths include all deaths for which stroke (ICD-10 I61–I64) is recorded as the underlying cause of death; stroke events comprise all stroke admissions and sudden stroke deaths; fatal strokes include fatal stroke admissions and sudden stroke deaths.

a Country or state/province population size at 31 December 2018 or nearest data available.

b Number of days between hospital admission date and date of death for all fatal stroke admissions.

Q1, Quartile 1; Q3, Quartile 3.

There were 761 531 fatal strokes, with women on average 5 years older than men at the time of a fatal stroke. Fatal stroke admissions comprised just over half of fatal strokes in men in WA, New Zealand, and Ontario, whereas sudden stroke deaths were more common in England and BC (Supplementary Figure 6). In women, sudden stroke deaths were more common, ranging from 51.6% in Ontario to 62.9% in BC. The average time between fatal stroke admission and death was 8 days in men and women.

### Stroke event rates

Age-standardized stroke event rates were highest in New Zealand and England in men, and in New Zealand in women, and lowest in men and women in Ontario (Fig. 1 and Table 2). There were age-adjusted declines in stroke event rates in all jurisdictions, with the greatest reductions in Ontario in men (−1.93%/year, 95% CI −2.04, −1.82) and women (−2.51%/year, 95% CI −2.61, −2.40). The average annual reductions were generally greater in women than in men in each jurisdiction.

**Figure 1:**
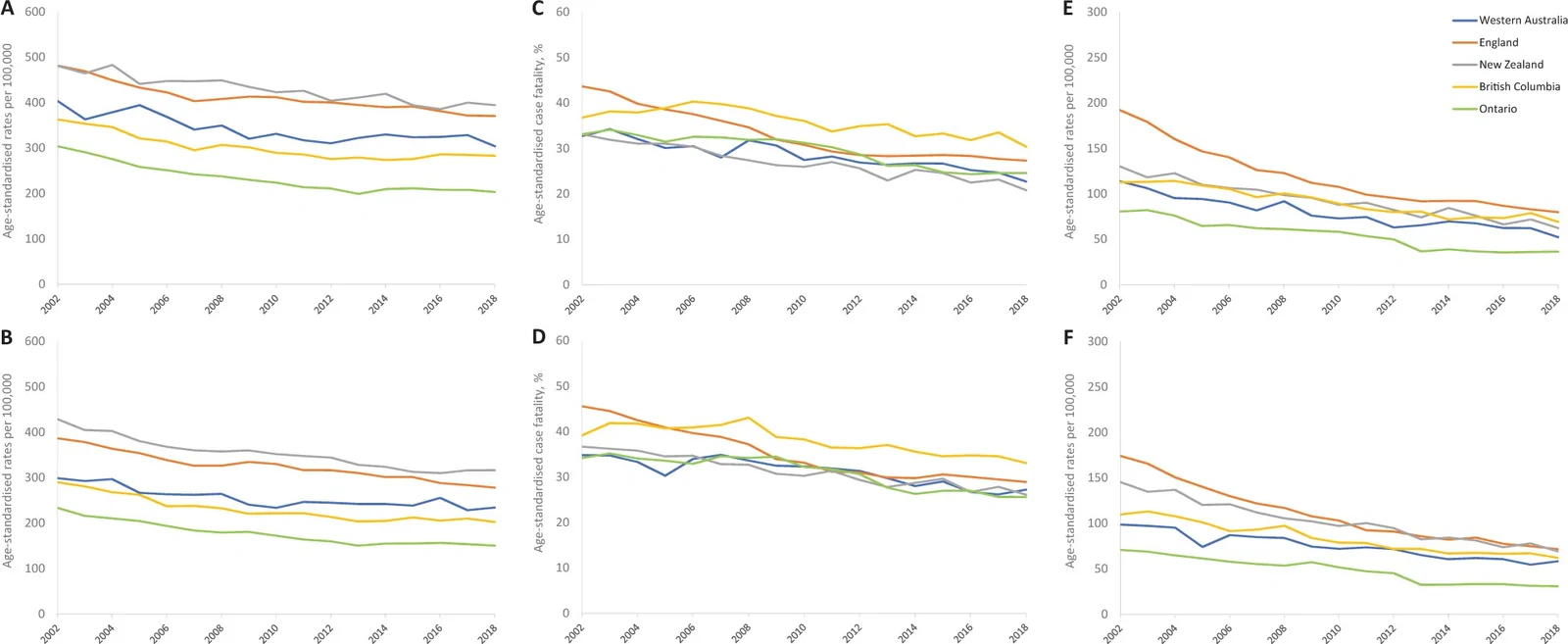
Age-standardized stroke events rates in (a) men and (b) women; age-standardized 30-day case fatality in (c) men and (d) women, and age-standardized stroke mortality rates in (e) men and (f) women, aged ≥35 years, 2002–18.

**Table 2:** Temporal trends in stroke event rates, 30-day case-fatality and mortality rates, and relative contribution to stroke mortality trends from trends in event rates and 30-day case fatality, by sex and jurisdiction, in people aged ≥35 years, 2002–18.

	Stroke event rate^a^			Thirty-day case fatality^b^			Stroke mortality rate^a^				Contribution to mortality decline [% (95% CI)]	
	2002	2018	Average annual change [% (95% CI)]^c^	2002	2018	Average annual change [% (95% CI)]^c^	2002	2018	Average annual change [% (95% CI)]^c^	Est. annual trend (%)^d^	Event rate	Case fatality
Men												
WA	403.3	304.0	−0.96 (−1.22, −0.70)	32.7	22.7	−3.28 (−3.90, −2.65)	113.9	52.3	−4.02 (−4.61, −3.43)	−4.24	22.6 (16.9, 28.8)	77.4 (71.2, 83.1)
England	481.6	370.5	−1.20 (−1.43, −0.98)	43.7	27.3	−5.09 (−5.19, −5.00)	192.2	80.0	−5.38 (−5.61, −5.15)	−6.30	19.1 (18.5, 19.8)	80.9 (80.2, 81.5)
NZ	480.7	394.5	−1.28 (−1.44, −1.12)	33.1	20.8	−3.66 (−4.04, −3.27)	130.3	62.5	−4.37 (−4.72, −4.01)	−4.90	25.9 (22.8, 29.2)	74.1 (70.8, 77.2)
BC	363.0	282.9	−1.24 (−1.42, −1.06)	36.8	30.4	−2.63 (−3.03, −2.24)	112.7	69.1	−3.30 (−3.65, −2.96)	−3.87	32.0 (27.6, 36.8)	68.0 (63.2, 72.4)
Ontario	303.8	203.3	−1.93 (−2.04, −1.82)	33.1	24.6	−2.23 (−2.39, −2.06)	80.5	36.4	−5.53 (−5.77, −5.30)	−5.30	36.5 (34.3, 38.6)	63.5 (61.4, 65.7)
Women												
WA	299.0	234.4	−1.32 (−1.59, −1.05)	34.8	27.2	−2.77 (−3.36, −2.18)	98.6	58.4	−3.50 (−3.99, −3.01)	−4.10	32.4 (26.3, 39.2)	67.6 (60.8, 73.7)
England	386.7	278.1	−1.91 (−2.09, −1.74)	45.5	28.9	−5.20 (−5.29, −5.11)	174.1	71.7	−5.54 (−5.72, −5.37)	−7.11	26.9 (26.3, 27.4)	73.1 (72.6, 73.7)
NZ	429.1	316.4	−1.77 (−1.92, −1.61)	36.7	26.1	−3.39 (−3.73, −3.05)	145.3	69.3	−4.28 (−4.56, −4.00)	−5.20	34.3 (31.3, 37.4)	65.7 (62.6, 68.7)
BC	289.9	202.6	−2.09 (−2.27, −1.91)	39.2	33.1	−2.60 (−2.98, −2.22)	109.8	62.1	−3.82 (−4.11, −3.53)	−4.69	44.7 (40.7, 49.0)	55.3 (51.0, 59.3)
Ontario	233.9	150.6	−2.51 (−2.61, −2.40)	34.2	25.6	−1.86 (−1.99, −1.72)	70.8	30.8	−5.39 (−5.59, −5.19)	−5.70	43.6 (41.7, 45.8)	56.4 (54.2, 58.3)

a Rates are standardized to the age distribution of the 2013 European Standard Population. Presented per 100 000 person-years.

b Thirty-day case fatality is standardized to the age distribution of all stroke events in each jurisdiction and is presented as a percentage.

c Represents the average annual percentage change, calculated from the exponential of the β-coefficient for calendar year (continuous variable) from Poisson regression models (event and mortality rates) and generalized binomial models with a logit-link function (30-day case fatality).

d Calculated as the sum of the average annual change in event rates and 30-day case fatality for each jurisdiction, stratified by sex.

Est., estimated; NZ, New Zealand.

### Thirty-day case fatality

Age-standardized 30-day case fatality was highest in England in 2002 (men, 43.7%; women, 45.5%), while, in other jurisdictions, the case-fatality estimates ranged from 32.7% to 36.8% in men and 34.2% to 39.2% in women (Table 2). There were age-adjusted declines in case fatality across all jurisdictions, with the greatest reduction in England in men (−5.09%/year, 95% CI −5.19, −5.00) and women (−5.20%/year, 95% CI −5.29, −5.11). Downward trends were generally driven by greater reductions in sudden stroke deaths compared with fatal stroke admissions, except in BC, where sudden stroke deaths remained higher than those in other jurisdictions (Supplementary Figure 7).

### Stroke mortality rates

Annual age-standardized stroke mortality rates were highest in England throughout most of the study period (Fig. 1 and  Table 2). The lowest mortality rates were in Ontario, declining from 80.5/100 000 to 36.4/100 000 in men and 70.8/100 000 to 30.8/100 000 in women. Age-adjusted annual declines in mortality rates in all jurisdictions ranged from −3.30%/year in BC to −5.53% in Ontario in men and from −3.5%/year in WA to −5.54%/year in England in women.

### Age-specific rates and trends

Stroke event and mortality rates and 30-day case fatality increased exponentially with increasing age in men and women, with higher case fatality in women than in men at most ages (Fig. 2 and  Supplementary Figure 8). The greatest reductions in stroke event rates were in those aged ≥75 years (∼2–3%/year), while event rates increased in those aged 35–54 years in all jurisdictions. Among people aged 55–64 years, event rates were generally unchanged except for increases in WA and BC (Supplementary Table 1). There were declines in 30-day case fatality in most age/sex groupings. Similarly, stroke mortality rates fell in men and women aged ≥55 years, while declines in stroke mortality in those aged 35–54 years were limited, with low rates in all jurisdictions (<10/100 000 person-years).

**Figure 2:**
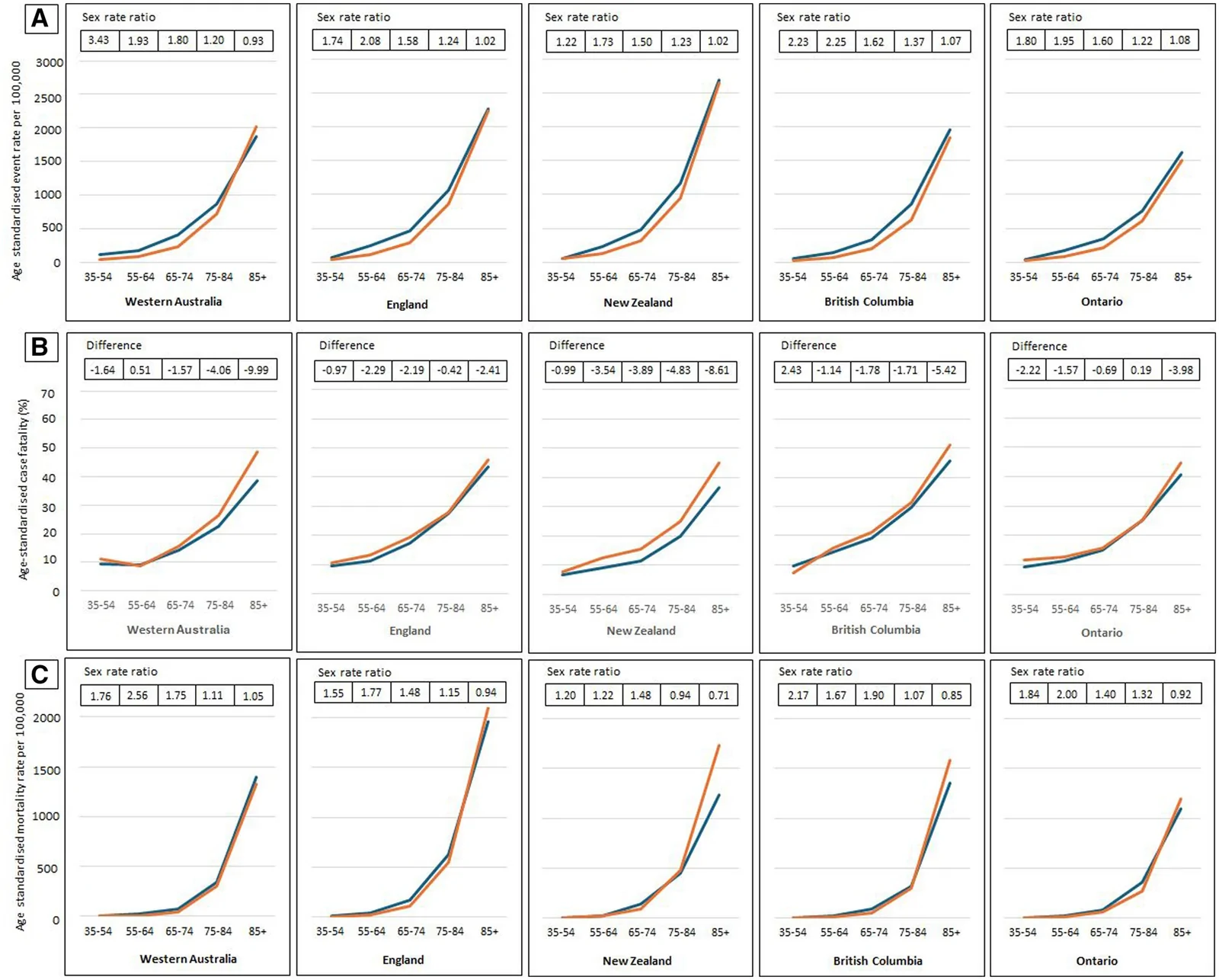
Age- and sex-specific (a) stroke event rates, (b) 30-day case fatality, and (c) stroke mortality rates in people aged ≥35 years in 2018.^a^  ^a^The sex rate ratio represents the ratio of age-standardized rates in men versus women in each age group, for stroke event and mortality rates. For 30-day case fatality, the difference is calculated by subtracting the age-standardized case fatality in women from men for each age group.

### Contribution of trends in event rates and case fatality

The declines in 30-day case fatality were a greater contributor than event rate trends to sex-specific reductions in stroke mortality (Table 2). In men, the contribution from declines in case fatality was ∼2/3 or more, with the lowest contributions in Ontario (63.5%, 95% CI 61.4, 65.7) and highest in England (80.9%, 95% CI 80.2, 81.5). In women, the contribution from declining case fatality was less than that in their male jurisdictional counterparts, ranging from 55.3% (95% CI 51.0, 59.3) in BC to 73.1% (95% CI 72.6, 73.7) in England.

Declines in stroke mortality in men aged <65 years and women aged <55 years were completely due to reductions in 30-day case fatality (Fig. 3). In women aged 55–64 years, there was a small contribution to downward stroke mortality from declining stroke event rates in New Zealand (30.6%), BC (15.6%), and Ontario (10.7%). In age groups of ≥65 years, there was a greater contribution of trends in case fatality, with similar patterns by sex (interaction *P* = .87) and a marginal difference between jurisdictions (interaction *P* = .049).

**Figure 3:**
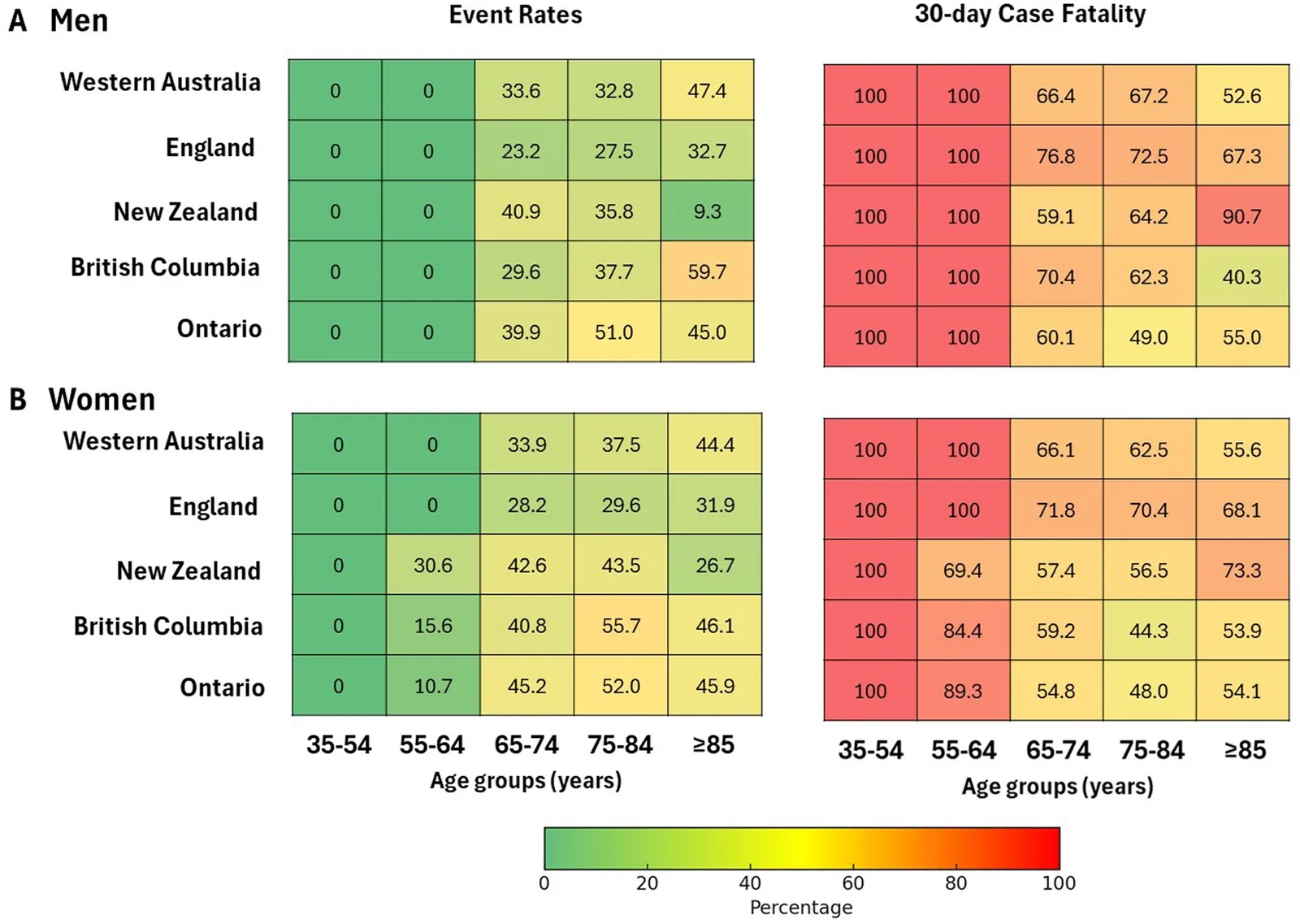
Age- and sex-specific relative contribution to stroke mortality rate trends from trends in stroke event rates and 30-day case fatality, stratified by jurisdiction, 2002–18.^a^^a^Numbers shown are percentages and represent the percentage contribution of trends in each of event rates and 30-day case fatality to trends in stroke mortality rates. Where trends in event rates and case fatality are opposing, the relative contributions are shown as zero and 100% for ease of interpretation.

### Sensitivity analyses

We tested the use of multiple-coded causes in defining stroke deaths. This resulted in 1.5–2 times higher stroke mortality rates in WA, BC, and England, and a smaller between-country differential in mortality rates compared with those in the main analyses, with limited impact on the identification of stroke deaths in New Zealand (Supplementary Table 2). Compared with the main analyses, the use of multiple-coded causes of death resulted in 3%–9% lower contributions from reductions in 30-day case fatality in WA, England, and BC (Supplementary Table 3), underpinned by smaller contributions in older men and women (Supplementary Figure 9).

## Discussion

The key finding of this study was that temporal improvements in 30-day case fatality made a greater contribution to declines in stroke mortality than falls in stroke event rates across all jurisdictions, with some between-country heterogeneity in the magnitude of the contribution. Contributions from trends in case fatality were generally higher in men than in women. While stroke event rates declined in older people, rates were unchanged or increasing in those aged 35–64 years. In younger age groups, reductions in stroke mortality were completely (in men) or almost completely (in women) underpinned by improvements in early survival post-stroke. Stroke event rates were higher in men except in the oldest age group and 30-day case fatality was generally higher in women across all ages.

Between 1990 and 2021, the Global Burden of Disease (GBD) reported a 40% reduction in stroke mortality globally, with average annual declines of 1%–2%/year for the countries included in our study [1]. In contrast, annual declines in stroke mortality in our study ranged from 3% to 5%/year, aligning with those reported from national statistics [16–18] and in part indicating greater reductions in mortality in the current era. We demonstrated some between-country variation in mortality rates, with the highest rates in England. Within Canada, stroke mortality rates were higher in BC than in Ontario, contributed to by higher rates in those aged ≥85 years in BC and explained in part by the higher mean age in that province.

Reductions in stroke event rates and case fatality have been posited as key to ongoing improvements in stroke mortality [2]. The multinational WHO MONICA study demonstrated that declining stroke event rates and case fatality correlate with declining stroke mortality, although it did not directly measure the contribution of each [15]. One of the few studies to directly measure contributions showed that, between 2001 and 2010, 78% of the reduction in stroke mortality in men in England was due to improvements in 30-day case fatality, while, in women, the contribution was 66% [5]. Our measurement of the relative contributions has demonstrated a similar pattern across jurisdictions over a longer period, although these patterns could differ in middle- and low-income countries, given the known disparity in the availability of and access to evidence-based treatments [19]. While this pattern was shown in older age groups in our study, the limited reduction in event rates in those aged <65 years meant that improvements in 30-day case fatality were primarily responsible for reductions in stroke mortality in younger adults.

Whether our findings have continued since 2018 may have been impacted by the effects of the COVID-19 pandemic, given the differential effects on cardiovascular disease (CVD) event rates and healthcare access between countries [20]. Most high-income countries saw reductions in hospitalizations for CVD, including stroke, although the magnitude of change differed [21, 22]. This likely reflects changes in access to and use of health services rather than changes in underlying stroke epidemiology. Based on local and GBD data, overall stroke mortality rates have continued to decline [10, 23], with a recent study from BC demonstrating that the greater impact of declining stroke case fatality continued into 2022 [24].

### Reasons for findings

Advances in treatments that have improved early survival and disability associated with stroke may explain some of our findings [10]. The availability and accuracy of neuroimaging techniques for detecting and differentiating ischaemic and haemorrhagic strokes have increased early survival [25], including earlier access to CT and MRI [26]. All jurisdictions in our study have reported substantial improvements in the receipt of IV thrombolysis (IVT) [10, 27–29], with 10.4% (England), 11.0% (Australia), and 12.2% and 12.8% (Canada and New Zealand, respectively) of ischaemic stroke patients undergoing IVT in 2019–21 [19]. With the evidence base for endovascular thrombectomy (EVT) only occurring in the later years of our study, the proportion of eligible stroke patients undergoing EVT varied from 2.4% in the UK to 7.2% and 10.0% in New Zealand and Australia, respectively [19].

However, higher stroke rates and poorer outcomes for Indigenous peoples, different ethnicities, and rural and remote communities are associated with structural healthcare inequities, systemic racism, and geographical disparities [30–32]. Lower access to reperfusion therapies in regional and rural areas could lead to differentials in stroke mortality within jurisdictional populations. Improved prehospital care, including thrombolysis and greater propensity to admit to hospital, could contribute to the reductions in sudden stroke deaths. The challenging geography and centralized stroke services in jurisdictions such as BC could influence their higher levels of sudden stroke deaths, given the association of geographical and temporal access with prehospital mortality [33].

Reductions in stroke event rates may be partially due to improvements in diagnostic accuracy, with lower misclassification of stroke mimics, although this is countered by increasing stroke incidence associated with the shift from transient ischaemic attack (TIA) to stroke due to changes in diagnostic criteria [11]. However, the lower contribution of falling stroke event rates in our study suggests that improvements in modifiable stroke risk factors may have been exceeded by improvements in the acute treatment of stroke. While the treatment and control of hypertension have improved in high-income countries, there has been less improvement in age-standardized hypertension prevalence in recent decades [34]. The largest increase in population-attributable fraction for stroke during 1990–21 was for body mass index and high ambient temperature. While the greatest contribution in high-income countries came from metabolic and behavioural risk factors, the stroke burden in low-income countries was more strongly associated with environmental factors [1].

There is increasing evidence of adverse trends in stroke rates in adults aged <55 years, consistently by stroke type and sex [13], aligning with those seen in MI and heart failure [35–37]. The shift from a symptom- and time-based definition to imaging-based criteria for differentiating stroke from TIA could contribute to an increase in event rates. One study reported increased ascertainment of stroke of 14.6% in those aged 18–55 years when new criteria were imposed [38], although similar or greater increases in ascertainment in older patients have also been reported [11]. Although temporal increases in population-attributable fractions for high body mass index (BMI), high ambient temperature, and diet-related factors have been reported, there is limited evidence of a cohort effect, particularly in high-income countries [39]. Our reporting of adverse trends in stroke event rates in those aged 55–64 years suggests a wider problem than has previously been described, with the potential to reverse low stroke mortality rates in these age groups.

The underlying cause of death has historically informed mortality statistics; however, some stroke deaths are under-recognized when this method is used [40, 41]—a general limitation of mortality statistics that are not specific to stroke. Conversely, there is an increasing focus on capturing the contributory causes of death, resulting in higher ascertainment of stroke deaths [42], associated with increasing multimorbidity [43]. Misclassification may be higher in stroke deaths that occur in the prehospital setting and this could have affected the temporal trends in stroke mortality. Changes to coding instructions have resulted in greater recording of vascular dementia as an underlying cause of death [44]. However, the implementation of standardized automated coding since 2013/14 in Australia, Canada, and England would have improved the between-country consistency of coding [45]. Our sensitivity analyses demonstrated that declines in stroke mortality were attenuated somewhat when the multiple-coded cause of death was used, although, despite this, improvements in case fatality remained a stronger driver of reductions in stroke mortality than event rates.

## Strengths and limitations

A key strength of this study was the use of standardized methods across the jurisdictions. Our use of whole-population data in each jurisdiction reduced the likelihood of selection bias and enabled the capture of prehospital and hospitalized stroke events. Between-country differences in stroke coding in hospital and mortality data could contribute to differences in rates and trends, particularly if coding standards change at different times. Our preliminary analyses of hospitalizations and deaths by type of stroke showed similar between-jurisdiction temporal patterns, indicating reasonable equivalence in stroke coding. The use of underlying cause of death may not perfectly align with true rates; however, our sensitivity analyses in which all cause-of-death fields were used demonstrated a limited impact on our overall conclusions. Stroke deaths occurring in the prehospital setting are more likely to be affected by misclassification; this limitation would apply to all jurisdictions in this study. We were unable to directly attribute improvements in 30-day case fatality to increasing use of specific therapies. While there may be some jurisdictional differences in prehospital care and the propensity to hospitalize patients, our measures capture the spectrum of hospitalized strokes and out-of-hospital deaths. Changes in diagnostic criteria have contributed to a higher proportion of hospitalized stroke events being of lower severity over time, although this definition has been implemented across all jurisdictions in our study. We were unable to present results stratified by type of stroke, as the differing temporal and jurisdictional patterns in hospitalized strokes coded as being of unspecified type may have imposed between-country biases.

## Conclusions

Stroke mortality continues to decline in high-income jurisdictions, driven by improvements in early mortality after stroke, likely reflecting investment in the early diagnosis and treatment of stroke patients. Further improvements in stroke burden could be achieved with additional targeting of vascular risk factors and addressing health-system and socio-economic barriers to the equitable prevention and treatment of stroke, which could lead to declines in avoidable stroke mortality. While the stroke mortality rates are relatively low in younger adults, there were concerning increases in stroke onset in people aged <65 years. Targeting prevention approaches to reduce stroke in this age group is essential to prevent future increases in the population burden of stroke and cardiovascular disease more broadly.

## Ethics approval

This study was approved by the University of Toronto Health Sciences Research Ethics Board, WA Department of Health Human Research Ethics Committee (2016/09/654), the New Zealand Health and Disability Ethics Committees (H13/049), and the Central and South Bristol Multi-Centre Research Ethics Committee (04/Q2006/176). As this work falls under the BC Centre for Disease Control’s mandate to monitor population health and its determinants, ethics board approval was not required.

## Supplementary Material

dyag157_Supplementary_Data

## Data Availability

Access to the datasets used in this study is generally not available due to agreements with the organizations providing the health data. No patient-level data were shared between jurisdictions. The base code used to generate the data for this study can be shared via a public repository. Access to the WA data was provided with the permission of data custodians to researchers named and approved by the WA Department of Health Human Research Ethics Committee. No additional data are available. Sharing of anonymized cohort data from New Zealand with other researchers or official agencies will generally be possible upon request from the authors (pending approval of the relevant official agencies). Access to the anonymized data used in this study was provided by Statistics NZ under the security and confidentiality provision of the Statistics Act 1975. Only people authorized by the New Zealand Statistics Act 1975 are allowed to see data about a particular person, household business, or organization. The Ontario dataset for this study is held securely in coded form at ICES. While legal data-sharing agreements between ICES and data providers (e.g. healthcare organizations and government) prohibit ICES from making the dataset publicly available, access may be granted to those who meet pre-specified criteria for confidential access. English national Hospital Episode Statistics and linked mortality data may be obtained by successful application to NHS England (www.digital.nhs.uk). Data-sharing agreements between NHS England and the Nuffield Department of Population Health prohibit the further sharing of any data other than statistical aggregates. Population Data BC (PopData BC) provided the de-identified linked administrative health datasets used in this study. Access to data provided by the Data Stewards is subject to approval but can be requested through the Data Stewards or their designated service providers. The datasets generated and/or analysed in the current study are not publicly available, to protect against any re-identification risk, but they may be made available upon reasonable request.
