# Supplementary material for: The impact of temporal trends in stroke event rates and 30-day case fatality on trends in stroke mortality: an international comparative study
Source: Int J Epidemiol. 2026 Sep 18;55(5):dyag157. doi: 10.1093/ije/dyag157 (PMC13653606; doi:10.1093/ije/dyag157)
Supplement: Supplementary file 1 [file dyag157_supplementary_data.docx]

**SUPPLEMENTARY DATA**

[**Supplementary File S1. Comparability of patterns of stroke coding between jurisdictions.**	2](#_Toc234585307)

[**Supplementary Figure S1.** Type of stroke as a proportion of all stroke hospitalisations* by year of admission, separately by jurisdiction: A. Western Australia; B. England; C. New Zealand; D. British Columbia; E. Ontario	3](#_Toc234585308)

[**Supplementary Figure S2.** Number of stroke deaths (ICD-10 I60-I64) stratified by underlying versus multiple-coded cause of death, in (A) Western Australia, (B) England, (C) New Zealand, (D) British Columbia and (E) Ontario^*^.	4](#_Toc234585309)

[**Supplementary Figure S3.** Composition of all stroke deaths based on underlying cause of death stratified by type of stroke and year of death in (A) Western Australia, (B) England, (C) New Zealand, (D) British Columbia and (E) Ontario.	5](#_Toc234585310)

[**Supplementary Figure S4.** Flow chart of definitions of stroke events.	6](#_Toc234585311)

[**Supplementary Figure S5.** Age distribution of total stroke events (2002 to 2018 combined), stratified by 5-year age group and jurisdiction.	7](#_Toc234585312)

[**Supplementary Figure S6.** Fatal strokes in (A) men and (B) women in 2002 and 2018, stratified as sudden stroke deaths and fatal stroke admissions.^*^	8](#_Toc234585313)

[**Supplementary Figure S7.** Age-standardised 30-day case fatality in men stratified by (A) sudden stroke deaths and (B) fatal stroke admissions; and in women (C) sudden stroke deaths and (D) fatal stroke admissions.	9](#_Toc234585314)

[**Supplementary Figure S8.** Age- and sex-specific (A) stroke event rates, (B) 30-day case fatality, and (C) stroke mortality rates in people aged ≥35 years in 2002 and 2012.	11](#_Toc234585315)

[**Supplementary Figure S9.** Sensitivity analysis for age- and sex-specific relative contribution to trends in stroke mortality from trends in stroke event rates and 30-day case fatality, stratified by jurisdiction, 2002 to 2018. Stroke deaths are identified using multiple-coded cause of death fields.	13](#_Toc234585316)

[**Supplementary Table S1.** Trends in stroke event rates, 30-day case fatality and mortality rates by jurisdiction, age, and sex, 2002 to 2018.	14](#_Toc234585317)

[**Supplementary Table S2.** Age-standardised stroke mortality rate ratios comparing rates calculated using stroke as an underlying cause of death only, versus stroke deaths identified from multiple-coded cause of death fields.	17](#_Toc234585318)

[**Supplementary Table S3.** Sensitivity analysis where stroke deaths were identified using multiple-coded cause of death fields, showing temporal trends in stroke event rates, 30-day case fatality and mortality rates, and contribution to stroke mortality trends of event rates and 30-day case fatality, by sex and jurisdiction, in people aged ≥35 years, 2002 to 2018.	18](#_Toc234585319)

### **Supplementary File S1. Comparability of patterns of stroke coding between jurisdictions.**

We assessed comparability of data between countries to inform study parameters by investigating patterns in coding of type of stroke in hospital and mortality data. This was undertaken in the broader study datasets in ≥20year olds, prior to stroke type and age restriction.

We found generally similar patterns and trends in the distribution of type of stroke in acute stroke hospital admissions, with the majority of strokes coded as cerebral infarction (International Classification of Diseases (ICD)-10 I63; 55%-60%) (Supplementary Figure 1). In all countries, the proportion of stroke hospitalisations coded as unspecified type (I64) declined over time, with low levels of use in the latter years of the period (<10% across jurisdictions).

We also assessed the difference between using underlying versus multiple-coded cause of death fields on our mortality definition. We found that using all cause of death fields (underlying and multiple-coded causes) to identify a stroke death increased stroke death numbers by 32% in Western Australia, 17% in England and 28% in British Columbia, with some differences over time (Supplementary Figure 2). In Ontario, multiple-coded cause of death coding was only available from 2013 onwards, where it increased stroke death numbers by 32%; in New Zealand, we found that coding of stroke as an associated cause of death was uncommon. As approximately 80% of deaths coded as cerebrovascular disease were classified as acute stroke, we excluded deaths coded as I65-I69 (data not shown). Of the acute stroke deaths, the majority were coded as unspecified stroke (I64), ranging from an annual average of 54.1% in New Zealand to 69.6% in British Columbia, although with temporal reductions over time.

### **Supplementary Figure S1.** Type of stroke as a proportion of all stroke hospitalisations* by year of admission, separately by jurisdiction: A. Western Australia; B. England; C. New Zealand; D. British Columbia; E. Ontario

*Stroke hospitalisations are identified from the principal discharge diagnosis field in people aged ≥20years. Inter- and intra-hospital transfers are accounted for and are therefore not double counted.

SAH, Subarachnoid haemorrhage; ICH, Intracerebral haemorrhage; N-ICH, Non-traumatic intracranial haemorrhage.

### **Supplementary Figure S2.** Number of stroke deaths (ICD-10 I60-I64) stratified by underlying versus multiple-coded cause of death, in (A) Western Australia, (B) England, (C) New Zealand, (D) British Columbia and (E) Ontario^*^.

A.

Number of deaths

|  | Underlying COD | Any COD |
| --- | --- | --- |
| 2002 | 710 | 1310 |
| 2003 | 703 | 1394 |
| 2004 | 695 | 1424 |
| 2005 | 592 | 1282 |
| 2006 | 702 | 1356 |
| 2007 | 662 | 1336 |
| 2008 | 743 | 1441 |
| 2009 | 656 | 1441 |
| 2010 | 656 | 1392 |
| 2011 | 679 | 1287 |
| 2012 | 647 | 1313 |
| 2013 | 648 | 1369 |
| 2014 | 662 | 1380 |
| 2015 | 688 | 1312 |
| 2016 | 703 | 1421 |
| 2017 | 692 | 1383 |
| 2018 | 697 | 1332 |

B.

Number of deaths

|  | Underlying COD | Any COD |
| --- | --- | --- |
| 2002 | 47714 | 61025 |
| 2003 | 45471 | 58664 |
| 2004 | 41324 | 53781 |
| 2005 | 38699 | 51142 |
| 2006 | 36776 | 48988 |
| 2007 | 34521 | 46570 |
| 2008 | 33527 | 46032 |
| 2009 | 31451 | 43922 |
| 2010 | 30641 | 43535 |
| 2011 | 28523 | 40861 |
| 2012 | 28446 | 41627 |
| 2013 | 27596 | 41349 |
| 2014 | 27590 | 41775 |
| 2015 | 28375 | 43092 |
| 2016 | 26915 | 42250 |
| 2017 | 26375 | 41655 |
| 2018 | 25904 | 41613 |
| 2019 | 25332 | 40821 |
| 2020 | 24878 | 44408 |
| 2021 | 24841 | 42949 |

C.

Nmber of deaths

|  | Underlying COD | Any COD |
| --- | --- | --- |
| 2002 | 2289 | 2289 |
| 2003 | 2151 | 2157 |
| 2004 | 2247 | 2250 |
| 2005 | 2094 | 2097 |
| 2006 | 2091 | 2097 |
| 2007 | 2070 | 2076 |
| 2008 | 2028 | 2028 |
| 2009 | 1962 | 1962 |
| 2010 | 1947 | 1953 |
| 2011 | 2058 | 2070 |
| 2012 | 1962 | 1974 |
| 2013 | 1743 | 1752 |
| 2014 | 1929 | 1950 |
| 2015 | 1878 | 1890 |
| 2016 | 1725 | 1821 |
| 2017 | 1845 | 1923 |
| 2018 | 1743 | 1809 |

D.

Number of deaths

|  | Underlying COD | Any COD |
| --- | --- | --- |
| 2002 | 1832 | 3107 |
| 2003 | 1923 | 3139 |
| 2004 | 1927 | 3150 |
| 2005 | 1893 | 3189 |
| 2006 | 1862 | 3177 |
| 2007 | 1893 | 3245 |
| 2008 | 2006 | 3406 |
| 2009 | 1865 | 3143 |
| 2010 | 1842 | 3134 |
| 2011 | 1836 | 3218 |
| 2012 | 1784 | 3183 |
| 2013 | 1816 | 3291 |
| 2014 | 1743 | 3283 |
| 2015 | 1803 | 3450 |
| 2016 | 1812 | 3580 |
| 2017 | 1960 | 3747 |
| 2018 | 1858 | 3483 |
| 2019 | 1878 | 3491 |
| 2020 | 1893 | 3626 |
| 2021 | 1914 | 3675 |

E.

Number of deaths

|  | Underlying COD | Any COD |
| --- | --- | --- |
| 2002 | 4538 | 4538 |
| 2003 | 4589 | 4589 |
| 2004 | 4434 | 4434 |
| 2005 | 4256 | 4256 |
| 2006 | 4248 | 4248 |
| 2007 | 4215 | 4215 |
| 2008 | 4183 | 4183 |
| 2009 | 4477 | 4477 |
| 2010 | 4364 | 4364 |
| 2011 | 4122 | 4122 |
| 2012 | 4085 | 4085 |
| 2013 | 3033 | 6298 |
| 2014 | 3192 | 6469 |
| 2015 | 3254 | 6505 |
| 2016 | 3353 | 6393 |
| 2017 | 3386 | 6314 |

*Associated cause of death coding was not available from Ontario prior to 2013, hence data from 2002 to 2012 represent underlying cause of death only.

COD, cause of death.

### **Supplementary Figure S3.** Composition of all stroke deaths based on underlying cause of death stratified by type of stroke and year of death in (A) Western Australia, (B) England, (C) New Zealand, (D) British Columbia and (E) Ontario.

SAH, Subarachnoid haemorrhage; ICH, Intracerebral haemorrhage; N-ICH, Non-traumatic intracranial haemorrhage.

### **Supplementary Figure S4.** Flow chart of definitions of stroke events.

Data confidentiality restrictions in some jurisdictions mean that numbers of events are suppressed where

counts are very small (<5), therefore there are some minor inconsistencies in numbers in some categories.

### **Supplementary Figure S5.** Age distribution of total stroke events (2002 to 2018 combined), stratified by 5-year age group and jurisdiction.

Percentage

| Age group, years | Western Australia | England | New Zealand | British Columbia | Ontario |
| --- | --- | --- | --- | --- | --- |
| 35-39 | 0.977491 | 0.650351 | 0.622267 | 0.531154 | 0.604181 |
| 40-44 | 1.603 | 1.16301 | 1.24694 | 0.970694 | 1.1931 |
| 45-49 | 2.70155 | 1.97882 | 2.08544 | 1.66963 | 2.19337 |
| 50-54 | 3.65662 | 3.06071 | 3.40445 | 2.86988 | 3.61653 |
| 55-59 | 5.08699 | 4.27817 | 4.69944 | 4.206 | 5.24703 |
| 60-64 | 6.66084 | 5.8171 | 6.41247 | 5.89622 | 6.98699 |
| 65-69 | 8.76379 | 7.92394 | 8.56518 | 7.95701 | 8.96519 |
| 70-74 | 10.9788 | 11.2251 | 11.5011 | 10.4162 | 11.5271 |
| 75-79 | 14.566 | 15.1409 | 14.944 | 13.8368 | 14.9366 |
| 80-84 | 16.9088 | 18.3889 | 17.9256 | 17.5168 | 17.7025 |
| 85-89 | 15.5636 | 16.8862 | 16.448 | 17.4612 | 15.2449 |
| 90-94 | 9.29737 | 10.0473 | 9.37725 | 11.7976 | 8.75337 |
| 95-99 | 2.77778 | 3.06014 | 2.44582 | 4.13085 | 2.63338 |
| 100 | 0.457358 | 0.379407 | 0.321945 | 0.740115 | 0.395715 |

### **Supplementary Figure S6.** Fatal strokes in (A) men and (B) women in 2002 and 2018, stratified as sudden stroke deaths and fatal stroke admissions.^*^

|  | Sudden stroke death | Fatal stroke admission |
| --- | --- | --- |
| Western Australia 2002 | 167 | 174 |
| Western Australia 2018 | 140 | 176 |
| England 2002 | 11150 | 7696 |
| England 2018 | 6736 | 5777 |
| New Zealand 2002 | 465 | 336.402 |
| New Zealand 2018 | 459 | 440.598 |
| British Columbia 2002 | 426 | 438 |
| British Columbia 2018 | 541 | 409 |
| Ontario 2002 | 1063 | 1222 |
| Ontario 2018 | 857 | 1111 |

|  | Sudden stroke death | Fatal stroke admission |
| --- | --- | --- |
| Western Australia 2002 | 245 | 210 |
| Western Australia 2018 | 218 | 241 |
| England 2002 | 18639 | 11048 |
| England 2018 | 8345 | 7525 |
| New Zealand 2002 | 465 | 459 |
| New Zealand 2018 | 597 | 597 |
| British Columbia 2002 | 770 | 430 |
| British Columbia 2018 | 714 | 432 |
| Ontario 2002 | 1601 | 1519 |
| Ontario 2018 | 1185 | 1229 |

*Stroke deaths were classified as a *sudden stroke death* where there was no stroke admission in the 30-days prior to the date of death. *Fatal stroke admissions* were those where death from any cause occurred within 30 days of a stroke admission.

### **Supplementary Figure S7.** Age-standardised 30-day case fatality in men stratified by (A) sudden stroke deaths and (B) fatal stroke admissions; and in women (C) sudden stroke deaths and (D) fatal stroke admissions.

*Stroke deaths were classified as a *sudden stroke death* where there was no stroke admission in the 30-days prior to the date of death. Fatal stroke admissions were those where death from any cause occurred within 30 days of a stroke admission.

### **Supplementary Figure S8.** Age- and sex-specific (A) stroke event rates, (B) 30-day case fatality, and (C) stroke mortality rates in people aged ≥35 years in 2002 and 2012.

**2002**

**2012**

### **Supplementary Figure S9.** Sensitivity analysis for age- and sex-specific relative contribution to trends in stroke mortality from trends in stroke event rates and 30-day case fatality, stratified by jurisdiction, 2002 to 2018. Stroke deaths are identified using multiple-coded cause of death fields.

*Numbers shown are percentages and represent the percentage contribution of trends in each of event rates and 30-day case fatality to trends in stroke mortality rates. Where trends in event rates and case fatality are opposing, the relative contributions are shown as zero and 100% for ease of interpretation.

### **Supplementary Table S1.** Trends in stroke event rates, 30-day case fatality and mortality rates by jurisdiction, age, and sex, 2002 to 2018.

|  | **Stroke event rate^†^** | | | | **30-day case fatality**^‡^ | | | | **Stroke mortality rate^†^** | | | | **Contribution to mortality decline,**  **% (95% CI)*** | | | |
| --- | --- | --- | --- | --- | --- | --- | --- | --- | --- | --- | --- | --- | --- | --- | --- | --- |
| **Age group (years)** | **2002** | **2018** | **Annual % change**  **(95% CI)** |  | **2002** | **2018** | **Annual % change (95% CI)** |  | **2002** | **2018** | **Annual % change**  **(95% CI)** | **Est. annual trend, %** | |  | **Event rate** | **Case fatality** |
| **Men** |  |  |  |  |  |  |  |  |  |  |  |  | |  |  |  |
| **Western Australia** | |  |  |  |  |  |  |  |  |  |  |  | |  |  |  |
| 35-54 | 39.1 | 54.2 | 2.78 (1.94, 3.62) |  | 16.61 | 9.64 | -4.38 (-6.86, -1.83) |  | 5.0 | 4.0 | -1.19 (-4.14, 1.85) | -1.6 | |  | 0 | 100 |
| 55-64 | 149.9 | 173.0 | 1.03 (0.34, 1.73) |  | 17.31 | 9.20 | -2.32 (-4.43, -0.17) |  | 20.8 | 12.4 | -1.05 (-3.43, 1.39) | -1.3 | |  | 0 | 100 |
| 65-74 | 490.1 | 409.3 | -1.31 (-1.84, -0.77) |  | 19.70 | 14.30 | -2.59 (-4.00, -1.17) |  | 69.1 | 49.0 | -3.45 (-4.99, -1.90) | -3.9 | |  | 33.6 (20.6, 53.8) | 66.4 (46.2, 79.4) |
| 75-84 | 1247.3 | 858.5 | -1.98 (-2.45, -1.52) |  | 35.01 | 22.56 | -4.05 (-5.05, -3.04) |  | 341.2 | 148.5 | -4.67 (-5.63, -3.70) | -6.0 | |  | 32.8 (25.7, 40.9) | 67.2 (59.1, 74.3) |
| ≥85 | 3191.6 | 1871.9 | -2.47 (-3.06, -1.86) |  | 50.94 | 38.58 | -2.73 (-3.94, -1.51) |  | 1395.0 | 556.4 | -4.40 (-5.34, -3.45) | -5.2 | |  | 47.4 (36.1, 62.9) | 52.6 (37.1, 63.9) |
| **England** |  |  |  |  |  |  |  |  |  |  |  |  | |  |  |  |
| 35-54 | 48.6 | 67.0 | 2.20 (2.01, 2.40) |  | 19.31 | 9.67 | -5.35 (-5.79, -4.91) |  | 7.7 | 5.3 | -2.52 (-3.02, -2.02) | -3.1 | |  | 0 | 100 |
| 55-64 | 214.0 | 236.2 | 1.01 (0.62, 1.40) |  | 21.81 | 11.05 | -5.08 (-5.40, -4.75) |  | 40.9 | 21.1 | -3.74 (-4.51, -2.96) | -4.1 | |  | 0 | 100 |
| 65-74 | 604.7 | 456.3 | -1.53 (-1.77, -1.29) |  | 31.45 | 17.18 | -5.09 (-5.30, -4.88) |  | 168.8 | 65.8 | -5.91 (-6.45, -5.36) | -6.6 | |  | 23.2 (21.9, 24.5) | 76.8 (75.5, 78.1) |
| 75-84 | 1531.2 | 1065.9 | -2.05 (-2.25, -1.84) |  | 46.05 | 27.42 | -5.40 (-5.56, -5.25) |  | 633.2 | 242.2 | -6.00 (-6.49, -5.51) | -7.4 | |  | 27.5 (26.6, 28.4) | 72.5 (71.6, 73.4) |
| ≥85 | 3424.0 | 2275.1 | -2.23 (-2.45, -2.01) |  | 62.28 | 43.53 | -4.59 (-4.78, -4.39) |  | 1966.9 | 849.3 | -4.99 (-5.38, -4.59) | -6.8 | |  | 32.7 (31.4, 34.1) | 67.3 (65.9, 68.6) |
| **New Zealand** | |  |  |  |  |  |  |  |  |  |  |  | |  |  |  |
| 35-54 | 49.3 | 55.0 | 0.86 (0.30, 1.43) |  | 11.90 | 6.56 | -2.79 (-4.58, -0.97) |  | 4.4 | 1.6 | -2.06 (-4.09, 0.02) | -5.1 | |  | 0 | 100 |
| 55-64 | 222.7 | 227.1 | 0.01 (-0.42, 0.45) |  | 12.86 | 8.96 | -2.61 (-3.88, -1.31) |  | 22.4 | 14.8 | -2.56 (-3.97, -1.13) | -5.2 | |  | 0 | 100 |
| 65-74 | 747.2 | 475.1 | -2.27 (-2.59, -1.95) |  | 21.47 | 11.50 | -3.27 (-4.12, -2.42) |  | 136.4 | 40.4 | -5.84 (-6.72, -4.96) | -9.1 | |  | 40.9 (34.5, 49.1) | 59.1 (50.9, 65.5) |
| 75-84 | 1573.3 | 1162.8 | -2.09 (-2.37, -1.82) |  | 34.53 | 20.01 | -3.74 (-4.36, -3.12) |  | 446.5 | 168.4 | -5.29 (-5.87, -4.70) | -9.0 | |  | 35.8 (31.2, 40.9) | 64.2 (59.1, 68.8) |
| ≥85 | 2642.0 | 2698.2 | -0.44 (-0.81, -0.07) |  | 52.96 | 36.49 | -4.33 (-5.06, -3.59) |  | 1232.3 | 798.9 | -3.26 (-3.86, -2.66) | -7.6 | |  | 9.3 (1.6, 16.3) | 90.7 (83.7, 98.4) |
| **British Columbia** | |  |  |  |  |  |  |  |  |  |  |  | |  |  |  |
| 35-54 | 31.16 | 45.0 | 1.51 (0.84, 2.17) |  | 22.54 | 9.73 | -4.86 (-6.65, -3.03) |  | 4.9 | 3.2 | -2.75 (-4.76, -0.70) | -3.3 | |  | 0 | 100 |
| 55-64 | 143.0 | 146.7 | 0.81 (0.30, 1.33) |  | 15.7 | 14.4 | -2.61 (-3.92, -1.28) |  | 18.2 | 16.2 | -1.38 (-2.81, 0.07) | -1.8 | |  | 0 | 100 |
| 65-74 | 432.5 | 325.1 | -1.26 (-1.63, -0.88) |  | 27.3 | 19.2 | -2.97 (-3.84, -2.10) |  | 92.2 | 49.5 | -3.47 (-4.33, -2.60) | -4.2 | |  | 29.6 (21.3, 39.4) | 70.4 (60.6, 78.7) |
| 75-84 | 1102.8 | 853.5 | -1.75 (-2.07, -1.44) |  | 36.2 | 29.6 | -2.90 (-3.55, -2.25) |  | 316.8 | 213.9 | -3.54 (-4.12, -2.96) | -4.6 | |  | 37.7 (31.2, 45.1) | 62.3 (54.9, 68.8) |
| ≥85 | 2964.5 | 1968.4 | -2.53 (-2.90, -2.17) |  | 51.4 | 45.7 | -1.71 (-2.44, -0.97) |  | 1349.1 | 777.5 | -3.35 (-3.89, -2.82) | -4.0 | |  | 59.7 (50.1, 72.4) | 40.3 (27.6, 49.9) |
| **Ontario** |  |  |  |  |  |  |  |  |  |  |  |  | |  |  |  |
| 35-54 | 36.9 | 44.0 | 2.24 (1.88, 2.61) |  | 15.6 | 9.4 | -4.24 (-5.31, -3.16) |  | 4.2 | 2.4 | -3.59 (-4.83, -2.33) | -2.0 | |  | 0 | 100 |
| 55-64 | 189.2 | 171.3 | -0.02 (-0.3, 0.26) |  | 15.8 | 11.2 | -3.08 (-3.85, -2.30) |  | 21.6 | 11.4 | -4.43 (-5.30, -3.56) | -3.1 | |  | 0 | 100 |
| 65-74 | 492.5 | 344.1 | -2.20 (-2.42, -1.99) |  | 22.0 | 15.1 | -3.32 (-3.85, -2.79) |  | 81.1 | 35.2 | -5.89 (-6.44, -5.32) | -5.5 | |  | 39.9 (35.6, 44.6) | 60.1 (55.4, 64.4) |
| 75-84 | 1379.9 | 754.3 | -3.24 (-3.43, -3.05) |  | 34.4 | 25.5 | -3.12 (-3.52, -2.71) |  | 361.2 | 138.8 | -5.85 (-6.23, -5.46) | -6.4 | |  | 51.0 (47.0, 55.7) | 49.0 (44.3, 53.0) |
| ≥85 | 2524.9 | 1625.7 | -3.04 (-3.29, -2.79) |  | 52.8 | 40.9 | -3.73 (-4.23, -3.23) |  | 1094.3 | 551.7 | -5.46 (-5.86, -5.06) | -6.8 | |  | 45.0 (41.1, 49.0) | 55.0 (51.0, 58.9) |
| **Women** |  |  |  |  |  |  |  |  |  |  |  |  | |  |  |  |
| **Western Australia** | |  |  |  |  |  |  |  |  |  |  |  | |  |  |  |
| 35-54 | 24.4 | 30.9 | 2.20 (1.14, 3.28) |  | 15.8 | 11.3 | -3.42 (-6.66, -0.06) |  | 2.8 | 2.9 | 0.56 (-3.38, 4.66) | -1.2 | |  | 0 | 100 |
| 55-64 | 85.5 | 89.5 | 0.67 (-0.34, 1.68) |  | 13.5 | 8.7 | -4.23 (-7.01, -1.36) |  | 8.2 | 5.4 | -4.73 (-8.00, -1.34) | -3.6 | |  | 0 | 100 |
| 65-74 | 294.6 | 227.2 | -1.11 (-1.80, -0.43) |  | 19.4 | 15.9 | -2.17 (-3.89, -0.42) |  | 39.5 | 23.0 | -3.56 (-5.41, -1.66) | -3.3 | |  | 33.9 (14.7, 73.5) | 66.1 (26.5, 85.3) |
| 75-84 | 1013.7 | 715.0 | -1.92 (-2.40, -1.45) |  | 35.6 | 26.6 | -3.21 (-4.20, -2.21) |  | 306.5 | 152.3 | -4.59 (-5.52, -3.65) | -5.1 | |  | 37.5 (28.7, 48.0) | 62.5 (52.0, 71.3) |
| ≥85 | 2602.3 | 2019.1 | -1.92 (-2.35, -1.49) |  | 59.5 | 48.6 | -2.40 (-3.28, -1.51) |  | 1330.7 | 848.9 | -3.08 (-3.70, -2.45) | -4.3 | |  | 44.4 (34.6, 57.0) | 55.6 (43.0, 65.4) |
| **England** |  |  |  |  |  |  |  |  |  |  |  |  | |  |  |  |
| 35-54 | 29.8 | 38.4 | 1.92 (1.63, 2.21) |  | 20.8 | 10.6 | -5.28 (-5.84, -4.72) |  | 5.0 | 3.1 | -2.96 (-3.57, -2.35) | -3.4 | |  | 0 | 100 |
| 55-64 | 111.4 | 113.4 | 0.64 (0.27, 1.01) |  | 23.8 | 13.3 | -4.63 (-5.05, -4.20) |  | 23.2 | 12.0 | -3.70 (-4.46, -2.93) | -4.0 | |  | 0 | 100 |
| 65-74 | 396.7 | 289.2 | -1.87 (-2.13, -1.62) |  | 32.0 | 19.4 | -4.78 (-5.02, -4.53) |  | 113.9 | 47.5 | -5.85 (-6.42, -5.27) | -6.6 | |  | 28.2 (26.7, 29.7) | 71.8 (70.3, 73.3) |
| 75-84 | 1274.2 | 857.2 | -2.33 (-2.49, -2.17) |  | 47.3 | 27.8 | -5.54 (-5.68, -5.40) |  | 548.6 | 203.8 | -6.23 (-6.55, -5.91) | -7.9 | |  | 29.6 (28.7, 30.4) | 70.4 (69.6, 71.3) |
| ≥85 | 3411.6 | 2233.7 | -2.39 (-2.57, -2.21) |  | 65.7 | 45.9 | -5.09 (-5.22, -4.97) |  | 2100.7 | 897.0 | -5.25 (-5.54, -4.95) | -7.5 | |  | 31.9 (31.1, 32.7) | 68.1 (67.3, 68.9) |
| **New Zealand** | |  |  |  |  |  |  |  |  |  |  |  | |  |  |  |
| 35-54 | 38.3 | 45.2 | 0.58 (-0.07, 1.22) |  | 13.6 | 7.5 | -3.68 (-5.61, -1.71) |  | 3.6 | 0.0 | -3.52 (-5.72, -1.26) | -11.1 | |  | 0 | 100 |
| 55-64 | 142.1 | 131.0 | -0.69 (-1.23, -0.14) |  | 16.5 | 12.5 | -1.55 (-3.13, 0.05) |  | 18.4 | 9.2 | -2.72 (-4.48, -0.93) | -4.4 | |  | 30.6 (6.2, 100.1) | 69.4 (0, 93.8) |
| 65-74 | 476.8 | 316.2 | -2.56 (-2.94, -2.18) |  | 24.3 | 15.4 | -3.44 (-4.39, -2.48) |  | 92.3 | 37.9 | -5.76 (-6.72, -4.79) | -9.1 | |  | 42.6 (35.6, 51.4) | 57.4 (48.6, 64.4) |
| 75-84 | 1517.5 | 943.3 | -2.60 (-2.87, -2.33) |  | 36.6 | 24.8 | -3.37 (-3.95, -2.79) |  | 473.6 | 177.4 | -5.58 (-6.10, -5.06) | -8.9 | |  | 43.5 (38.9, 48.7) | 56.5 (51.3, 61.1) |
| ≥85 | 3210.6 | 2651.2 | -1.30 (-1.56, -1.05) |  | 59.1 | 45.1 | -3.57 (-4.09, -3.06) |  | 1735.9 | 997.2 | -3.51 (-3.87, -3.13) | -7.1 | |  | 26.7 (22.0, 31.6) | 73.3 (68.4, 78.0) |
| **British Columbia** | |  |  |  |  |  |  |  |  |  |  |  | |  |  |  |
| 35-54 | 14.6 | 20.2 | 2.17 (1.26, 3.09) |  | 21.1 | 7.3 | -2.45 (-4.84, 0) |  | 2.3 | 1.1 | 0.89 (-1.91, 3.76) | -0.3 | |  | 0 | 100 |
| 55-64 | 76.5 | 65.3 | -0.71 (-1.4, -0.01) |  | 18.7 | 15.6 | -3.85 (-5.58, -2.09) |  | 10.9 | 5.6 | -4.48 (-6.33, -2.59) | -4.6 | |  | 15.6 (-0.1, 31.9) | 84.4 (68.1, 100.1) |
| 65-74 | 270.2 | 200.5 | -1.66 (-2.12, -1.19) |  | 22.6 | 21.0 | -2.4 (-3.47, -1.32) |  | 48.6 | 33.0 | -3.64 (-4.69, -2.58) | -4.1 | |  | 40.8 (29.6, 56.7) | 59.2 (43.3, 70.4) |
| 75-84 | 924.5 | 624.6 | -2.54 (-2.86, -2.23) |  | 38.1 | 31.3 | -2.02 (-2.67, -1.37) |  | 296.6 | 165.8 | -3.93 (-4.49, -3.37) | -4.6 | |  | 55.7 (47.8, 65.3) | 44.3 (34.7, 52.2) |
| ≥85 | 2872.4 | 1844.5 | -2.53 (-2.81, -2.26) |  | 58.6 | 51.1 | -2.96 (-3.52, -2.40) |  | 1579.8 | 884.2 | -3.85 (-4.22, -3.48) | -5.5 | |  | 46.1 (41.1, 52.0) | 53.9 (48.0, 58.9) |
| **Ontario** |  |  |  |  |  |  |  |  |  |  |  |  | |  |  |  |
| 35-54 | 21.5 | 24.5 | 1.73 (1.27, 2.20) |  | 17.3 | 11.6 | -4.68 (-5.99, -3.36) |  | 2.3 | 1.3 | -3.45 (-5.00, -1.88) | -2.95 | |  | 0 | 100 |
| 55-64 | 99.4 | 87.9 | -0.3 (-0.68, 0.08) |  | 16.5 | 12.8 | -2.53 (-3.53, -1.52) |  | 10.8 | 6.7 | -4.28 (-5.41, -3.12) | -2.83 | |  | 10.7 (-3.0, 23.7) | 89.3 (76.3, 100.0) |
| 65-74 | 346.3 | 215.3 | -2.72 (-2.98, -2.47) |  | 23.0 | 15.8 | -3.30 (-3.92, -2.68) |  | 58.1 | 22.4 | -5.93 (-6.57, -5.29) | -6.02 | |  | 45.2 (40.3, 50.9) | 54.8 (49.1, 59.7) |
| 75-84 | 1036.1 | 616.3 | -3.24 (-3.43, -3.06) |  | 33.7 | 25.0 | -2.99 (-3.38, -2.60) |  | 273.0 | 118.0 | -5.89 (-6.25, -5.53) | -6.23 | |  | 52.0 (48.7, 55.8) | 48.0 (44.2, 51.3) |
| ≥85 | 2458.8 | 1507.2 | -2.89 (-3.07, -2.71) |  | 56.0 | 43.2 | -3.41 (-3.76, -3.06) |  | 1190.4 | 516.0 | -5.14 (-5.41, -4.87) | -6.30 | |  | 45.9 (42.9, 49.0) | 54.1 (51.0, 57.1) |

† Rates are standardised to the age distribution of the 2013 European Standard Population and presented per 100,000 person-years.

‡30-day case fatality is standardised to the age distribution of all stroke events within each jurisdiction.

*Calculated as the average annual change for stroke events and 30-day case fatality, divided by the estimated annual trend in stroke mortality.

CI, confidence interval.

### **Supplementary Table S2.** Age-standardised stroke mortality rate ratios comparing rates calculated using stroke as an underlying cause of death only, versus stroke deaths identified from multiple-coded cause of death fields.

|  | **Mortality Rate Ratio** | | | | |
| --- | --- | --- | --- | --- | --- |
|  | **Western Australia** | **England** | **New Zealand** | **British Columbia** | **Ontario*** |
| Men |  |  |  |  |  |
| 2002 | 1.9 | 1.3 | 1.0 | 1.9 | - |
| 2003 | 2.1 | 1.3 | 1.0 | 1.8 | - |
| 2004 | 2.4 | 1.3 | 1.0 | 1.8 | - |
| 2005 | 2.4 | 1.3 | 1.0 | 1.9 | - |
| 2006 | 2.2 | 1.3 | 1.0 | 1.9 | - |
| 2007 | 2.2 | 1.4 | 1.0 | 2.0 | - |
| 2008 | 2.1 | 1.4 | 1.0 | 1.9 | - |
| 2009 | 2.4 | 1.4 | 1.0 | 1.8 | - |
| 2010 | 2.4 | 1.4 | 1.0 | 1.9 | - |
| 2011 | 2.0 | 1.4 | 1.0 | 1.9 | - |
| 2012 | 2.2 | 1.4 | 1.0 | 2.0 | - |
| 2013 | 2.4 | 1.4 | 1.0 | 2.0 | 1.3 |
| 2014 | 2.2 | 1.4 | 1.0 | 2.1 | 1.3 |
| 2015 | 2.1 | 1.4 | 1.0 | 2.1 | 1.3 |
| 2016 | 2.3 | 1.3 | 1.0 | 2.2 | 1.2 |
| 2017 | 2.2 | 1.3 | 1.0 | 2.1 | 1.3 |
| 2018 | 2.3 | 1.3 | 1.0 | 2.1 | 1.3 |
| Women |  |  |  |  |  |
| 2002 | 1.9 | 1.3 | 1.0 | 1.7 | - |
| 2003 | 1.9 | 1.3 | 1.0 | 1.6 | - |
| 2004 | 2.0 | 1.3 | 1.0 | 1.7 | - |
| 2005 | 2.0 | 1.3 | 1.0 | 1.7 | - |
| 2006 | 1.9 | 1.3 | 1.0 | 1.7 | - |
| 2007 | 1.9 | 1.3 | 1.0 | 1.7 | - |
| 2008 | 2.0 | 1.3 | 1.0 | 1.7 | - |
| 2009 | 2.1 | 1.4 | 1.0 | 1.7 | - |
| 2010 | 2.1 | 1.4 | 1.0 | 1.7 | - |
| 2011 | 1.9 | 1.4 | 1.0 | 1.7 | - |
| 2012 | 1.9 | 1.4 | 1.0 | 1.8 | - |
| 2013 | 2.1 | 1.4 | 1.0 | 1.8 | 1.3 |
| 2014 | 2.1 | 1.4 | 1.0 | 1.9 | 1.3 |
| 2015 | 1.9 | 1.4 | 1.0 | 1.9 | 1.3 |
| 2016 | 2.0 | 1.3 | 1.0 | 1.9 | 1.2 |
| 2017 | 1.9 | 1.3 | 1.0 | 1.9 | 1.2 |
| 2018 | 1.8 | 1.3 | 1.1 | 1.9 | 1.2 |

*Multiple-coded cause of death data was only available in Ontario from 2013 onwards.

### **Supplementary Table S3.** Sensitivity analysis where stroke deaths were identified using multiple-coded cause of death fields, showing temporal trends in stroke event rates, 30-day case fatality and mortality rates, and contribution to stroke mortality trends of event rates and 30-day case fatality, by sex and jurisdiction, in people aged ≥35 years, 2002 to 2018.

|  | **Stroke event rate†** | | | **30-day case fatality‡** | | | | | **Stroke mortality rate†** | | | | **Contribution to mortality decline, (%)*** | |
| --- | --- | --- | --- | --- | --- | --- | --- | --- | --- | --- | --- | --- | --- | --- |
|  | 2002 | 2018 | Average annual change, % (95% CI) |  | 2002 | 2018 | Average annual change, % (95% CI) |  | 2002 | 2018 | Average annual change, % (95% CI) | Est. annual trend (%) | Event rate | Case fatality |
| Men |  |  |  |  |  |  |  |  |  |  |  |  |  |  |
| WA | 486.6 | 357.8 | -1.40 (-1.64, -1.16) |  | 44.1 | 34.5 | -3.19 (-3.71, -2.67) |  | 216.1 | 118.0 | -3.76 (-4.15, -3.36) | -4.6 | 30.4 (25.6, 35.7) | 69.5 (64.2, 74.3) |
| England | 536.5 | 418.5 | -1.18 (-1.39, -0.96) |  | 50.0 | 36.3 | -4.04 (-4.13, -3.95) |  | 249.0 | 104.7 | -5.18 (-5.25, -5.11) | -5.2 | 22.6 (21.8, 23.3) | 77.4 (76.7, 78.2) |
| NZ | 480.2 | 397.1 | -1.24 (-1.40, -1.08) |  | 32.9 | 21.3 | -3.49 (-3.87, -3.10) |  | 130.3 | 64.7 | -4.09 (-4.44, -3.73) | -4.7 | 26.2 (23.0, 29.6) | 73.8 (70.4, 77.0) |
| BC | 446.4 | 352.2 | -1.12 (-1.28, -0.95) |  | 48.3 | 44.1 | -1.61 (-1.95, -1.27) |  | 212.6 | 148.2 | -2.17 (-2.41, -1.92) | -2.7 | 41.0 (35.0, 47.8) | 59.0 (52.0, 64.9) |
| Women |  |  |  |  |  |  |  |  |  |  |  |  |  |  |
| WA | 367.5 | 269.5 | -1.80 (-2.04, -1.56) |  | 46.5 | 35.9 | -3.18 (-3.69, -2.66) |  | 184.2 | 104.4 | -3.53 (-3.88, -3.18) | -5.0 | 36.1 (31.5, 41.5) | 63.8 (58.6, 68.6) |
| England | 427.0 | 311.4 | -1.82 (-1.98, -1.66) |  | 51.3 | 36.7 | -4.29 (-4.37, -4.21) |  | 222.1 | 93.1 | -5.18 (-5.24, -5.12) | -6.1 | 29.8 (29.2, 30.4) | 70.2 (69.6, 70.8) |
| NZ | 429.4 | 318.3 | -1.72 (-1.87, -1.56) |  | 36.19 | 26.23 | -3.23 (0, -2.89) |  | 144.6 | 73.1 | -4.05 (-4.33, -3.77) | -4.9 | 34.7 (31.7, 38.0) | 65.3 (62.1, 68.3) |
| BC | 356.2 | 250.1 | -1.94 (-2.10, -1.78) |  | 50.55 | 45.12 | -1.85 (-2.19, -1.52) |  | 187.5 | 117.4 | -2.78 (-3.00, -2.56) | -3.8 | 51.1 (46.6, 56.5) | 48.8 (43.4, 53.6) |

† Rates are standardised to the age distribution of the 2013 European Standard Population and presented per 100,000 person-years.

‡30-day case fatality is standardised to the age distribution of all stroke events across jurisdictions.

*Calculated as the average annual change for stroke events and 30-day case fatality, divided by the estimated annual trend in stroke mortality.

WA, Western Australia; NZ, New Zealand; BC, British Columbia.
